# Prevalence of common mental disorder and associated factors among pregnant women in South-East Ethiopia, 2017: a community based cross-sectional study

**DOI:** 10.1186/s12978-019-0834-2

**Published:** 2019-11-28

**Authors:** Ashenafi Mekonnen Woldetsadik, Abebaw Nigussie Ayele, Adem Esmael Roba, Genet Fikadu Haile, Khan Mubashir

**Affiliations:** 1 Department of Midwifery, Madda Walabu University, Goba Referral Hospital, School of Health Science, Bale Goba, Ethiopia; 2 Department of Physiology, Madda Walabu University, Goba Referral Hospital, School of Medicine, Bale Goba, Ethiopia; 3 Department of Nursing, Madda Walabu University, Goba Referral Hospital, School of Health Science, Bale Goba, Ethiopia; 4 Department of Midwifery, Madda Walabu University, Goba Referral Hospital, School of Health Science, Bale Goba, Ethiopia; 5 Department of Biochemistry, Madda Walabu University, Goba Referral Hospital, School of Medicine, Bale Goba, Ethiopia

**Keywords:** Common mental disorder, Pregnant mothers, Social support, South East Ethiopia, Forced sexual intercourse, Infant mortality

## Abstract

**Background:**

Mothers suffering from common mental disorder (CMD), such as anxiety and depression may not be able to function properly, which could adversely affect the mother-infant bond and even result in increased infant morbidity and mortality. The purpose of this study was to assess the prevalence of CMD and its determinants among pregnant women in Southeast Ethiopia.

**Methods:**

Data was collected from 743 pregnant women via interview-administered, standardised questionnaires during Dec–Jan 2017. The WHO Self-Reported Questionnaire (SRQ) was used to screen CMD. Multivariate logistic regression was conducted and ORs and 95% confidence intervals were calculated.

**Results:**

The prevalence of CMD during pregnancy was 35.8% (95% CI: 34–38%) and the main determinants of CMD were: illiteracy, presence of health risk, financial instability, physical or emotional abuse, having sexual intercourse without her willingness**,** family history of psychiatric illness and history of chronic medical illness.

**Conclusion:**

CMD prevalence during pregnancy was high, indicating a need to regularly screen pregnant women for CMD and its determinants as part of routine obstetric care.

## Plain English summary

Common mental disorder (CMD) like anxiety and depression are very common during pregnancy. Women with mental illness during pregnancy usually have poor physical health and may be associated with negative impact on child development. So this study was aimed to see the prevalence of CMD in pregnant women of southeast Ethiopia. We screened 743 pregnant women for CMD using the SRQ research tool developed by WHO. This study was community based and the women were asked questions during face to face interviews at their homes. The prevalence of CMD among the pregnant women was found to be 35.8%. Many factors like relationship problems, physical or emotional abuse, poor husband support, family history of psychiatric illness and history of chronic medical illness were found directly associated with CMD. Our study also revealed that the women who were forced for sexual intercourse, were illiterate, had pregnancy complications, or were having health risk and with financial instability had the likelihood of developing CMD.

## Background

Globally, around 450 million people are living with mental illness [1]. In low and lower middle income countries the non-psychotic perinatal common mental disorder (CMD) is common primarily among poorer women with gender-based risks or a psychiatric history [2]. From 2005 to 2009, out of 10, one pregnant women experienced at least one major depressive episode in one year [3]. In developing countries perinatal depression is common and one in three women had significant mental health disorder but is under-estimated public health concern in low and middle income countries making substantial contribution to maternal and infant morbidity and mortality [4]. In high-income countries perinatal mental illness is the leading cause of maternal morbidity and mortality and causes unfavorable impacts on short and long term physical and mental health of off springs [5, 6]. In low, middle and high income countries, the prevalence of CMD during pregnancy varies from 12 to 43% [7].

The main determinants of antenatal CMD are women’s marital status, unplanned pregnancy, gestational age and bleeding [4], Furthermore, the presence of poor health status before conception like headache, diabetic mellitus, hypertension, absence of support from partner, history of intimate partner violence and being from low socioeconomic status also leads to antenatal CMD [5, 6].

A number of independent studies have identified the association between antenatal depression and adverse neurobehavioral outcomes including reduced social, emotional and cognitive functioning during childhood development [8]. Besides depression, anxiety disorders, eating disorders and psychosis are the antenatal mental disorders which are associated with pre-term delivery, low birth weight of newborn, increased infant mortality and postnatal psychosis causing subsequent emotional problems in the child and adolescent [9].

Ethiopia is one of the low income countries with high rates of mental health problems in pregnant women ranging from 9.2–33% in different areas of the Ethiopia [4]. Even though some studies have been carried out in Ethiopia to recognize the impact of this issue, but none of them has focused on South Eastern region [10–12]. So the current study was carried out in the Bale zone of South-East Ethiopia. This zone is considered to be the developed and representative area with much better antenatal facility available in this region. So this study was intended to assess the prevalence of CMD and its associated factors during pregnancy in South-East Ethiopia.

## Material and methods

### Study design, population and sampling procedures

A community based cross-sectional study was conducted in three administrative towns (Robe, Goba and Ginnir) of Bale zone South-East Ethiopia from December–January 2017. The three administrative towns encompass a total of seven Kebeles (Kebele is the smallest administrative unit in Ethiopia similar to that of ward); out of which Robe has three, Goba and Ginnir each has two Kebeles. Using a simple random sampling technique, one Kebele from Goba (East Goba), one from Ginnir (01 Kebele) and two from Robe town (Café Donsa and Baha Biftu) were selected. In the selected 3 towns the number of pregnant women registered by health extension workers was 2376. Out of this, 1086 were found in Robe town, 840 and 450 in Goba and Ginnir towns respectively. The prevalence of CMD during pregnancy ranged from 9.2–33% in different areas of the Ethiopia [4, 10–12]. Hence, a single population proportion formula was used to obtain maximum sample size of 748. Multistage sampling technique was used to select study subjects. The calculated sample size was proportionally allocated based on the estimated number of pregnant women in selected Kebeles. Therefore, 341 from Robe, 265 from Goba and 142 pregnant women from Ginnir town were selected for the study. Then, the study participant was selected using systematic random sampling technique at every K^th^ interval; where K^th^ is the ratio of estimated number of pregnant women in each Kebele to the proportionally allocated sample size for specific Kebele. Therefore, we accessed the pregnant women at every 3rd interval. From the first three women, the third one was selected by lottery method. With the help of antenatal registration book maintained by the health extension workers the selected participants were located and interviewed in their homes. All registered pregnant women at any trimester living in the study area for at least six months were included in the study except those taking antidepressants and who had known mental health problems.

### Data collection tools and procedures

The data was collected by face to face interview using structured questionnaire addressing socio-demographic and obstetric characteristics of study participants which was developed after reviewing the literature. The List of threatening experience questionnaire (LTE-Q) [13] was adopted and modified in present context to assess the stressful life events. The Oslo-3 Social Support Scale (OSS) [14], the Abuse Assessment Screen (AAS) questions [15] and WHO’s alcohol, smoking and substance involvement screening test (WHO-ASSIST V3.0) [16] were used to assess social support, presence of victimization and substance or alcohol abuse, respectively. To screen CMD during pregnancy, the standardized WHO’s Self-Reporting Questionnaire (SRQ-20) was used and those who answered “Yes” to six or more of the twenty questions were categorized as CMD caseness (Yes, No). This criterion was validated by the study conducted in Butajira, Ethiopia [17]. Twelve trained data collectors and four supervisors were involved in the process.

### Data quality control

The questionnaires were translated from English into the local languages (Amharic and Afan Oromo) using language experts. To confirm that meaning was not altered in the translation process the translated questionnaires were tested for reliability and validity on 5% of the final sample size with pregnant women in Delomena, a town in Bale Zone that was not included in the study. The results obtained were found to be consistent with Amharic version, so there was no need to translate it back. Data collectors and supervisors received two days of training on proper instrument administration and study protocol. Throughout the data collection period, the supervisor monitored the data collectors and 10% of the collected daily data were checked by the field supervisors and principal investigator. Incomplete questionnaires were excluded from the study and counted as non-respondents.

### Data processing and analysis

Double data entry was performed for all data and then Epi Info 7.1.2’s validation program was used to check the completeness, accuracy and consistency of data and exported to statistical package for social sciences (SPSS) 21 for the analysis [18, 19]. Descriptive statistics were calculated to obtain percentages, frequencies and means for all variables. Those variables with significance level (*p*-value) < 0.05 in bivariate analysis were entered into multivariate logistic regression model for further analysis in order to adjust the confounding factor on the dependent variables.

## Results

The response rate of current study was 99.3%.

### Socio-demographic factors

The mean ± SD age of participants was 27.3 ± 5.2 with the majority being 20–34 years of age (83.6%). As shown in Tables 1, 68.5% were of the Oromo ethnic group and 43.7% were Muslims. The majority of participants worked as housewives (61.1%) and had either a primary or secondary school education (55.8%). Most participants were married (96.4%) and of these, over half of them had been married for more than 4 years (59.8%) (Table 1).

**Table 1:** Socio-demographic characteristics of the participants in Bale Zone South East Ethiopia, 2017 (*n* = 743)

Variables	Frequency	% of the total sample
Age		
≤ 19 years	36	4.8
20–34 years	621	83.6
≥ 35 years	86	11.6
Religious status		
Muslim	325	43.7
Orthodox	254	34.2
Other	164	22.1
Marital status		
Married	716	96.4
Others®	27	3.6
Ethnicity		
Oromo	509	68.5
Amhara	170	22.9
Others®®	64	8.6
Educational status		
Unable to read and write	105	14.1
Able to read and write	140	18.8
Primary school	220	29.6
Secondary school	196	26.4
Diploma and above	82	11.0
Number of years married		
≤ 1 year	87	11.7
2–4 years	212	28.5
≥ 5 years	444	59.8
Occupation		
Housewife	454	61.1
Employed	64	8.6
Merchant	77	10.4
Private Employee	71	9.6
Farmer	39	5.2
Unemployed**®®®**	38	5.1
Husband’s education		
Unable to read and write	74	10.0
Able to read and write	123	16.6
Primary school	163	21.9
Secondary school	246	33.1
Diploma	81	10.9
Degree and above	56	7.5
Family member indebted in the last six months		
Yes	78	10.5
No	665	89.5
Hunger within the last six months		
Yes	52	7.0
No	691	93.0
Family wealth in relation to others		
Low	235	31.6
Moderate	415	55.9
High	93	12.5

Note: **other**; protestant and catholic, **Others®**; Single, divorced, widowed and separated, **others®®**; Tigray, Gurage, Wolayita and Seltie, unemployed**®®®**, student and daily laborer

### Obstetric care factors

More than half of the participants (54.6%) were in the second trimester of their pregnancy and had been pregnant 2–4 times before (58.8%). In this sample, 21.4% had an unplanned pregnancy, 12.5% had history of abortion, and 4% had a prior history of a neonatal death. The participants who had at least one antenatal care (ANC) follow up for the current pregnancy accounted up to 90.8% (Table 2).

**Table 2:** Obstetric characteristics of participants in Bale Zone South East Ethiopia, 2017 (*n* = 743)

Variables	Frequency	% of the total sample
Pregnancy planned		
Yes	584	78.6
No	159	21.4
Total number of pregnancies		
	184	24.8
2–4	437	58.8
≥ 5	122	16.4
Gestational age of pregnancy		
First trimester	55	7.4
Second trimester	406	54.6
Third trimester	282	38.0
Number of alive children		
0	197	26.5
1–2	334	45.0
≥ 3	212	28.5
Past history of still birth		
Yes	51	6.9
No	692	93.1
History of abortion		
Yes	93	12.5
No	650	87.5
Previous history of neonatal death		
Yes	30	4.0
No	713	96.0
History of past pregnancy complication		
Yes	123	16.6
No	620	83.4
Complication with current pregnancy		
Yes	100	13.5
No	643	86.5
ANC follow-up for current pregnancy		
Yes	675	90.8
No	68	9.2

### Psychosocial factors

Amongst the participants, 14.4% had financial instability, 10.9% had legal problems, 8.1% had relationship issues, 7.9% had health risks, and 5.5% had lost a loved one. Seventy-seven (10.4%) reported that they had been emotionally or physically abused during their lifetime and 6.2% reported abuse during the current pregnancy. Thirty-four (4.6%) participants reported being forced to have sexual intercourse within the last year and of these, the majority (88.2%) reported that it was their husband who forced them for intercourse (Table 3).

**Table 3:** Psychosocial characteristics of participants in Bale Zone South East Ethiopia, 2017 (*n* = 743)

Variables	Frequency	% of the total sample	Relative %
Health risk			
Yes	59	7.9	
No	684	92.1	
Loss of loved one			
Yes	41	5.5	
No	702	94.5	
Financial stress			
Yes	107	14.4	
No	636	85.6	
Legal problem			
Yes	81	10.9	
No	662	89.1	
Relationship problem			
Yes	60	8.1	
No	683	91.9	
History of emotional or physical abuse			
Yes	77	10.4	
No	666	89.6	
Have you been abused during this pregnancy			
Yes	46	6.2	
No	697	93.8	
If you are abused during the current pregnancy, by whom			
Partner	43		93.5
Others	3		6.5
Forced sexual activities in last one year			
Yes	34	4.6	
No	709	95.4	
Perpetrator of forced sex			
Partner	30		88.2
Others®	4		11.8
On how many people do you rely in home			
> 5	306	41.2	
3–5	226	30.4	
1–3	179	24.1	
None	32	4.3	
Number of people showing interest in what you do			
A lot	241	32.4	
Some	177	23.8	
Uncertain	190	25.6	
Little	69	9.3	
None	66	8.9	
Participant’s neighbour			
Very difficult	52	7.0	
Difficult	117	15.7	
Possible	242	32.6	
Easy	212	28.5	
Very easy	120	16.2	
Husband’s support for the continuation of pregnancy			
Stronger	413	55.5	
Moderate	256	34.5	
Poor	74	10.0	
Family support for continuation of pregnancy			
Yes	553	74.4	
No	190	25.6	

Note: Other- family member, stranger Others®: family member, stranger

### Substance abuse

Most participants reported no substance abuse, 25.2% of the participants reported ever drinking alcoholic beverages and 10.4% reported ever chewing khat. Of those who drink alcohol, 46.5% reported having alcohol on a monthly basis (Table 4).

**Table 4:** Substance abuse by the participants in Bale Zone South East Ethiopia, 2017 (*n* = 743)

Variable	Frequency	% of the total sample	Relative %
Ever drank alcohol beverage			
Yes	187	25.2	
No	556	74.8	
How often drank alcohol beverage			
Once or twice	55		29.4
Monthly	87		46.5
Weekly	31		16.6
Daily	10		5.3
Almost daily	4		2.1
Ever used substance like khat			
Yes	77	10.4	
No	666	89.6	
How often have you used substance			
Once or twice	24		31.2
Monthly	26		33.8
Weekly	14		18.2
Daily	10		13.0
Almost daily	3		3.8

### Clinical factors

Participants were asked about various clinical conditions and forty-five (6.1%) reported to have a history of chronic medical illness and 10 (1.3%) had a history of psychiatric illness. Additionally, 58 (7.8%) had a family history of psychiatric illness.

### Common mental disorders

The SRQ-20 was used to assess the prevalence of common mental disorder amongst the participants. The mean score was 3.75 and 266 (35.8%; 95% CI: 34–38%) participants had total scores of 6 or higher suggesting that they were experiencing mental health problems during their pregnancy. The symptoms experienced most frequently were: tiring easily (*n* = 328, 44.2%), often having headaches (*n* = 238, 32.0%), having a poor appetite (*n* = 214, 28.8%) and experiencing uncomfortable feelings in the stomach (*n* = 168, 22.6%).

### Factors associated with common mental disorders during pregnancy

Adjusting for inability to read and write (AOR = 2.06; 95% CI: 1.05–4.04), health risks (AOR = 2.94; 95% CI: 1.53–5.66), financial instability (AOR = 1.72; 95% CI: 1.06–2.82), physical or emotional abuse (AOR = 2.40; 95% CI: 1.36–4.24), forced sexual intercourse in last one year (AOR = 3.85; 95% CI: 1.67–8.88), family history of psychiatric illness (AOR = 3.14; 95% CI: 1.66–5.94) and history of chronic medical illness (AOR = 3.26; 95% CI: 1.64–6.48) showed statistically significant association (*p* < 0.05) with CMD (Table 5).

**Table 5:** Factors associated with common mental disorder during pregnancy in participants in Bale Zone South East Ethiopia, 2017

Variables	CMD		Crude OR with 95% CI	Adjusted OR with 95% CI
	Yes	No		
Educational status				
Unable to read and write	57	48	2.87 (1.56–5.29)	2.06 (1.05–4.04)*
Read and write	49	91	1.30 (0.68–2.47)	1.30 (0.68–2.47)
Primary school	75	145	1.25 (0.72–2.17)	1.06 (0.58–1.93)
Secondary school	61	135	1.09 (0.62–1.92)	0.92 (0.50–1.69)
Diploma and above	24	58	1.00	1.00
History of abortion				
Yes	47	46	2.01 (1.30–3.12)	1.34 (0.80–2.34)
No	219	431	1.00	1.00
History of pregnancy complication in past				
Yes	65	58	2.34 (1.58–3.46)	1.56 (0.99–2.34)
No	201	419	1.00	1.00
Health risk				
Yes	222	457	4.65 (2.61–8.27)	2.94 (1.53–5.66)**
No	41	18	1.00	1.00
Loss of loved one				
Yes	23	18	2.41 (1.28–4.56)	1.93 (0.94–3.88)
No	243	459	1.00	1.00
Financial stress / instability				
Yes	65	42	3.35 (2.20–5.11)	1.72 (1.06–2.82)*
No	201	435	1.00	1.00
Relationship problem				
Yes	36	24	2.36 (1.48–3.76)	1.97 (0.98–3.66)
No	230	453	1.00	1.00
Ever physically or emotionally abused				
Yes	51	26	4.12 (2.50–6.78)	2.40 (1.36–4.24)**
No	215	451	1.00	1.00
Forced sexual activities in last one year				
Yes	24	10	4.63 (2.18–9.84)	3.85 (1.67–8.88)**
No	242	467	1.00	1.00
Husbands support				
Stronger	125	288	1.00	1.00
Moderate	92	164	1.29 (0.93–1.80)	0.93 (0.64–1.35)
Poor	49	25	4.52 (2.67–7.64)	1.07 (0.58–3.89)
Practical family support				
Yes	172	381	1.00	1.00
No	94	96	2.17 (1.55–3.04)	1.47 (0.98–2.21)
Family history of psychiatric illness				
Yes	38	20	3.81 (2.17–6.70)	3.14 (1.66–5.94)**
No	228	457	1.00	1.00
History of chronic medical illness				
Yes	28	17	3.18 (1.71–5.93)	3.26 (1.64–6.48)**
No	238	460	1.00	1.00

Note: *p value is significant at *p* < 0.05 **p value is significant at *p* < 0.01 1.00 = Reference for category

## Discussion

The overall prevalence of CMD during pregnancy from our study finding was 35.8% (95% CI: 34–38%), which is higher than the studies conducted in Maringa Parana (12.9%) [20], Brazil (20.2%) [21], Pakistan (18%) [22], Peru (30%), Vietnam (21%), India (30%) [23], Nigeria (7%) [24] and Kilimanjaro (28.8%) [25], and slightly lower than reported in Tanzania (39.5%) [26] and in the two Cape Town’s peri-urban settlements (39%) [27]. These differences in CMD prevalence might be attributed to differences in measurement tools used, level of knowledge and understanding of the participants, sample size and socio-cultural and economic variations. Similarly, within Ethiopia, there were also variations in CMD prevalence rates, all of which were lower than the current study; for example: Butajira (33%) [10], Maichew (31.1%) [11], Gondar University Hospital (23%) [28], Debre Tabor town (11.8%) [29] and Addis Ababa health facility (24.94%) [30]. These variations may be due to differences in sample size, the time of study, the age, location, and educational status of participants and/or the tools used to diagnose CMD.

In the present study the women who were not able to read and write were 2.08 times more likely to have CMD than the literate women. Our findings were in agreement with a population-based cohort study in Southern Brazil where lower educational levels were significantly associated with antenatal depressive manifestations [31]. However a study conducted in rural Bangladesh, India and Pakistan reported that being literate [32] and spending more than 10 years in formal education were pre-disposing factors for CMD during pregnancy [33]. In addition to socio-economic and cultural differences this discrepancy may be due to the personal circumstances like the relationship quality with the intimate partner, empowerment at home and society and the workload in the populations studied.

Furthermore, in our study, women having a history of previous pregnancy-related complications had 1.59 times chance of development of CMD during pregnancy. While in two different studies conducted in Sao Paulo [34] and Debre Tabor Town [29], women with current pregnancy-related complications were at risk of developing CMD. This is obvious, since during pregnancy a women feels depressive state of mind and any kind of complication during this period may make it worse.

The socio-economic factors like financial instability and history of physical and/or emotional abuse showed the likelihood of CMD 1.88 and 2.47 times respectively. Our results were consistent with previous researches carried out in Ethiopia and other countries where financial instability was found to predict the development of CMD during pregnancy [11, 33, 35, 36] as did a history of physical and/or emotional abuse [24, 27, 32, 33, 37–39]. and forced sexual activities over the past year [32, 33]. In addition to the basic needs of life, moral, social and financial support is important for the wellbeing of a person and lack of any of them may lead to mental instability. Similar to our current findings, the absence of husband’s support during pregnancy in two peri-urban settlements in Cape Town [27] were the main predictors of CMD. Also a family history of psychiatric illness was significantly associated with CMD during pregnancy in the present study.

Lastly, in this study, pregnant women with a history of chronic medical illness were vulnerable for CMD, which are similar with the findings of a study conducted in Brazil, Maringa and Parana [20]. Women with chronic illnesses are more worried about their sickness and also remain detached from the social life, hence causing a disturbed mental state. A major limitation of current study is the use of a standardized screening tool to measure CMDs but not locally validated. Further it is a cross-sectional study, hence a temporal relationship could not be determined; only an association between the variables, and not causation, could be inferred. In our study we addressed that there is a strong knowledge gap in the pregnant women about CMD. Further our study is a community based study it can be generalized to the population at large.

## Conclusion

The prevalence of self-reported CMD during pregnancy in Bale Zone south east Ethiopia is high. Being unable to read and write, a history of previous pregnancy-related complications, financial instability, a history of physical or emotional abuse, being forced to engage in sexual intercourse activities over the past one year, poor husband support, a family history of psychiatric illness and a history of chronic medical illness are the main predictors for CMD during pregnancy. The Bale Zone health office in collaboration with key stakeholders should organize the seminars, conferences and create the awareness programs in communities regarding CMD. Further the women education should be encouraged and for this they should be morally and socially supported.

## Data Availability

All the available data and material used in this study is presented in the main paper.

## Abbreviations

AAS: Abuse Assessment screen
ANC: Antenatal Care.
CMD: Common Mental Disorder
LTE-Q: List of Threatening experience questionnaire
OR: Odds Ratio
OSS: Oslo 3 social support scale
SPSS: Statistical package for Social Sciences
SRQ: Self Reported Questionnaire
WHO: World Health Organization
